# Repeated social defeat causes bone loss from mouse femur

**DOI:** 10.1016/j.bbih.2026.101295

**Published:** 2026-06-30

**Authors:** Do-Gyoon Kim, Farah A. Al-Omari, Natalie R. Gallagher, Jiyeon Kim, Jie Liu, Minji Kim, Olivia Jackson, John F. Sheridan, Beth S. Lee

**Affiliations:** a Division of Orthodontics, College of Dentistry, The Ohio State University, Columbus, OH, 43210, USA; b Division of Biosciences, College of Dentistry, The Ohio State University, Columbus, OH, USA; c Department of Orthodontics, Graduate School of Clinical Dentistry, Ewha Womans University, Seoul, Republic of Korea; d Department of Physiology and Cell Biology, College of Medicine, The Ohio State University, Columbus, OH, 43210, USA; e Department of Orthodontics, School of Dental Medicine, University of Colorado Anschutz Medical Campus, Aurora, CO, USA

**Keywords:** Repeated social stress, Femur, Micro-computed tomography, Characterization

## Abstract

**Introduction:**

Clinical studies have demonstrated that depression increases the risk of bone fracture and impaired bone growth. However, the mechanisms by which psychosocial stress aggravates bone properties remain to be fully elucidated. Repeated social defeat (RSD) is a murine model system that recapitulates key physiological, immunological, and behavioral alterations observed in humans exposed to repeated psychosocial stress. The objective of this study was to examine whether RSD alters bone characteristics and to explore the mechanisms underlying these effects.

**Methods:**

Twenty-four wild-type (WT) male C57BL/6N mice (6–8 weeks old) were obtained and assigned equally into control and stress groups. For the stress group, an aggressive CD-1 male mouse (12 months old) was introduced into the cage of an established cohort of three resident C57BL/6N mice for 2 h daily for 6 consecutive days. Seventeen hours after the last stress cycle, the spleen and femur were dissected. The femurs were then scanned using micro-computed tomography (micro-CT) to analyze bone volume, mineral content, morphological parameters, and growth plate thickness. Following non-destructive micro-CT scanning, the femurs were fractured to measure their strength. Biomarkers of osteoclast activity and splenomegaly were also assayed.

**Results:**

Spleen weight and osteoclastic biomarkers were significantly higher in the stress group than in the control group (p < 0.03). The stress group also showed significantly lower values of mineral, volume, and morphology of bone than the control group (p < 0.04). These bone changes resulted in weaker femur strength compared to the control group (p < 0.01). Additionally, the growth plate was significantly thinner in the stress group (p < 0.01) and contained disordered chondrocytes.

**Discussion:**

Repeated social defeat in adolescent male mice resulted in weakened bone characteristics and altered growth plate morphology. Increased biomarkers of osteoclast number and activity were observed in the stress group. These findings suggest that RSD can impair bone health and growth in mice, which may provide insight into how psychosocial stressors like bullying or low social status could similarly impact bone health in humans.

## Highlights

• Repeated social defeat (RSD) enhanced osteoclast activity, leading to bone loss.
• RSD inhibited periosteal bone formation in the femurs of adolescent mice.
• RSD contributed to decreased femoral fracture strength.
• The RSD mice group had thinner growth plates than the control mice group.

## 1 Introduction

In the United States, more than 50 million people have low bone mass (Wright et al., 2014). Anxiety and depression due to psychological stress are also prevalent disorders, as more than 10 million patients are diagnosed annually (Bab and Yirmiya, 2010; Blazer et al., 1994). Numerous studies have shown that psychological stress contributes to bone loss (Calarge et al., 2014; Kelly et al., 2019; Erez et al., 2012; Pedersen et al., 2016; Follis et al., 2019). Patients with depression face an increased risk of bone fractures (Calarge et al., 2014; Pedersen et al., 2016). However, the mechanisms by which psychological stress exacerbates bone loss remain unclear.

Some forms of mental stress have been shown to induce an immunosuppressed, low inflammation state (Reader et al., 2015; Liu et al., 2017; Sorrells et al., 2009; Blume et al., 2011; Dhabhar, 2009). In contrast, more recent studies have demonstrated that repeated psychosocial stresses, such as bullying or low socioeconomic status, result in an immunoenhanced, pro-inflammatory state (Reader et al., 2015; Niraula et al., 2018; Weber et al., 2017). The murine repeated social defeat (RSD) model is a stressor that recapitulates key physiological, immunological, and behavioral alterations observed in humans exposed to psychosocial stress (Reader et al., 2015; Weber et al., 2017). This model elevates levels of sympathetic-mediated release of catecholamines and corticosterone, along with pro-inflammatory cytokines that promote proliferation of myeloid lineage cells, including monocytes (Weber et al., 2017; Yin et al., 2019; Sawicki et al., 2019). These responses to RSD stimulate bone marrow hematopoiesis to promote monocytes that overexpress pro-inflammatory cytokines (Niraula et al., 2018, 2019; Weber et al., 2017; Yin et al., 2019; McKim et al., 2018a). These monocytes are released into circulation and are actively recruited to the brain, where they accumulate and trigger neuroinflammation associated with anxiety-like behavior (Weber et al., 2017; Niraula et al., 2019).

Notably, these monocytes are also the major precursor population of osteoclasts (bone resorbing cells) in the blood (Madel et al., 2019). However, no studies have been conducted to investigate the effects of RSD on bone loss. We hypothesized that RSD-induced monocytes could lead to increased osteoclast formation and activity, ultimately causing bone loss. Thus, the objective of this study was to examine whether RSD alters bone characteristics and to explore the mechanisms underlying these effects.

## 2 Materials and methods

### 2.1 Specimen preparation

All experiments were conducted in accordance with the NIH Guidelines for the Care and Use of Laboratory Animals and were approved by the Ohio State University Institutional Laboratory Animal Care and Use Committee. Twenty-four male C57BL/6N (6-8 weeks old) and CD-1 (12 months old) mice were obtained from Charles River Laboratories (Wilmington, Massachusetts). C57BL/6N mice were housed in cohorts of three, while CD-1 mice were singly housed in polypropylene cages at 21°C room temperature under a 12-h light/dark cycle with *ad libitum* access to water and rodent chow. Twelve mice were randomly selected for inclusion in experimental treatment groups. Repeated social defeat (RSD) stress was performed as previously described (Sawicki et al., 2019). In brief, an aggressive male intruder CD-1 mouse (12 months, retired breeder) was introduced into 4 cages of established male cohorts (3 per cage) of C57BL/6N mice for 2 h during 6 consecutive afternoons between 3 p.m. and 5 p.m. If the intruder did not initiate an attack within 5–10 min or was attacked by any of the resident mice, then a new intruder was introduced. At the end of the 2-h period, the intruder was removed and the residents were left undisturbed until the following day when the paradigm was repeated. Different intruders were used on consecutive days. The control 12 C57BL/6N mice (3 in a cage) were undisturbed. Wounding was scored before sacrifice using a scale from 0 to 5, where 0 indicated no visible or palpable wounds, and 5 indicated confluent wounds on the back and hind limbs (Yin et al., 2019).

Spleen, femora, and blood were collected following carbon dioxide asphyxiation, 17 h after the last cycle of stress. The mice were fasted for 6 h before blood collection via cardiac puncture. The spleen was weighed immediately after dissection and preserved in formalin solution. Femora were cut from each mouse, and the soft tissues were removed. These specimens were then wrapped in gauze soaked in normal saline and frozen at −20°C until needed.

### 2.2 Biomarkers of osteoclast-mediated bone resorption

Blood samples and wound scores from five mice in the control group and six mice in the RSD group were used to assess biomarkers. IL-6 levels were measured by ELISA (Abcam, Cambridge, UK). To assess osteoclast activity, serum levels of TRAP (tartrate-resistant acid phosphatase) and CTX-I (carboxyterminal cross-linking telopeptide of type I bone collagen) were quantified by immunoassay. Serum TRAP levels were measured using the MouseTRAP ELISA kit, and serum CTX-I levels were measured using the RatLaps EIA kit, both from Immunodiagnostic Systems (Qiagen N.V., Germany).

### 2.3 Micro-computed tomography (micro-CT)

The 24 femora (one from each mouse) were randomly selected and thawed at room temperature. Three femora from the control group broke during tissue harvesting and were excluded from the study. All remaining specimens were scanned using micro-CT (Scanco μCT50; SCANCO Medical AG, Brüttisellen, Switzerland) with a voxel size of 10 × 10 × 10 μm^3^, under identical scanning conditions of 70 kV, 114 μA, and 900 ms integration time.

Bone voxels were segmented from non-bone voxels using a heuristic algorithm to determine the whole bone (WB) region (Liu et al., 2022). The cortical bone (CB) and trabecular bone (TB) voxels were digitally separated using a compartmentalizing method. Briefly, the internal cavity, including TB and marrow, was masked to obtain the TB. Then, the CB was then isolated by removing the TB from the WB (Liu et al., 2022; Buie et al., 2007; Kim et al., 2012, 2018).

### 2.4 Volume, mineral density, and morphology of each bone region

Total volume (TV) was determined by multiplying the voxel size by the sum of the voxels of the masked internal cavity and WB. The CT attenuation value of each bone voxel was calibrated to tissue mineral density (TMD) using known densities of commercial hydroxyapatite (HA) phantoms (1220 and 1540 mgHA/cm^3^). Total mineral content (TMC) was computed by multiplying the total sum of TMD by the volume of WB (BV_WB_). Bone mineral density (BMD) was measured by dividing TMC by TV. Mean, standard deviation (SD), and lower and upper 5th percentiles (Low_5_ and High_5_) of a frequency plot of TMD were obtained for each region.

Histomorphometric parameters of the CB were measured in a region spanning 50 slices, centered at 55% of the femoral length from the head. Total area (Tt.Ar), including cortical bone and cavity, only cortical bone area (Ct.Ar), and their ratio (Ct.Ar/Tt.Ar) were measured. Thickness (Ct.Th), periosteal perimeter (Perimeter), and outer and inner diameters along the anterior-posterior and medial-lateral axes (DAP_outer, DAP_inner, DML_outer, and DML_inner), the outer diameter ratio (AP/ML), and minimum inertia (I_min_), were also obtained.

**Fig. 1:**
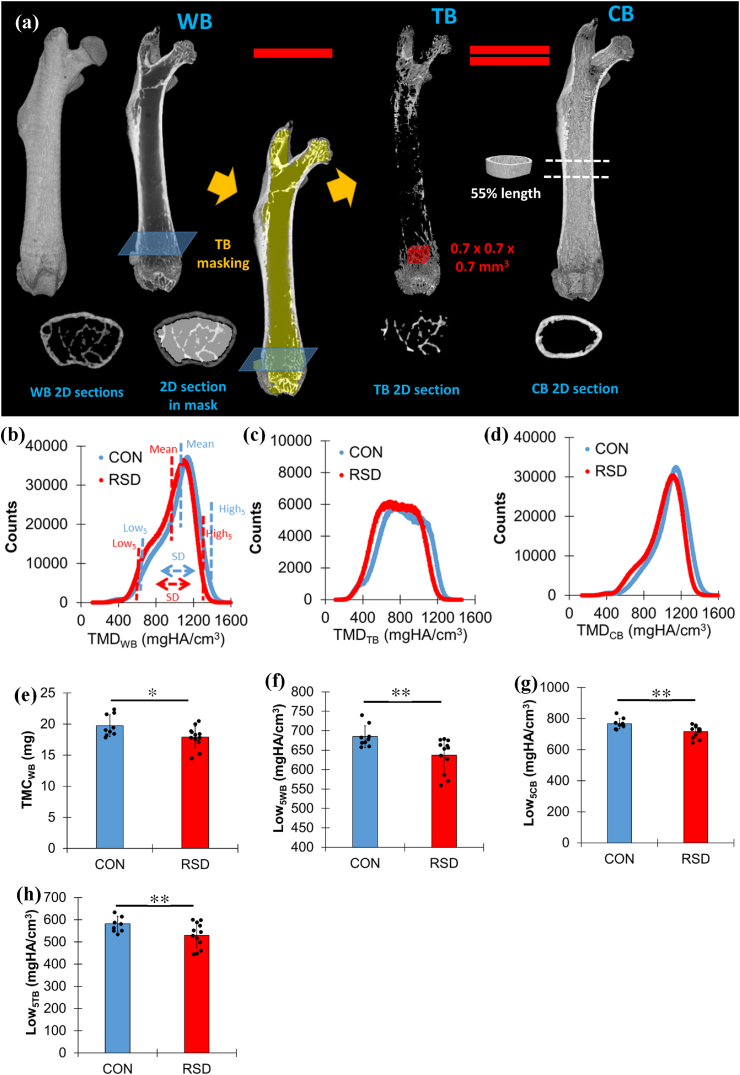
(a) Steps of compartmentation for the 3D micro-CT image of a femur. The whole bone (WB) was identified by digitally removing the outer soft tissue and inner bone marrow tissue. Masking was used to isolate the bone marrow cavity, including trabecular bone (TB), and to digitally separate the cortical bone (CB) from the TB. Tissue mineral density (TMD) parameters were determined in individual frequency plots of (b) WB, (c) CB, and (d) TB of control (CON) and stress (RSD) groups. Significantly different values of the parameters between control (CON) and stress (RSD) groups for (e) total mineral content (TMC_WB_), and 5th percentile low (Low_5WB_, Low_5CB_, Low_5TB_) of (f) the whole bone (WB), (g) cortical bone (CB), and (h) trabecular bone (TB). ∗; p < 0.05, ∗∗; p < 0.01.

Morphological parameters of the TB were determined using the same dimensions for the region of interest (0.70 × 0.70 × 0.70 mm^3^) located above the growth plate at the distal femoral condyle (Fig. 1a). Trabecular bone volume fraction (BV/TV_TB_), surface-to-volume ratio (BS/BV), and trabecular number (Tb.N), thickness (Tb.Th), and separation (Tb.Sp) were computed using the morphological code (CTAn, SkyScan, Kontich, Belgium).

### 2.5 Micro-CT based analysis of distal growth plate

The growth plate was identified at the distal metaphysis of femur using the micro-CT image. The bone region surrounding the growth plate was isolated by expanding the growth plate by up to 3 voxels (30 μm) in both upper and lower directions. 3D histomorphometric parameters of the growth plate including total volume (TV), bone volume (BV), bone fraction (BV/TV), surface area, mean thickness (Th.mean), and maximum thickness (Th.Max) were measured using ImageJ software (NIH). The TMD parameters of the growth plate bone region were also assessed.

### 2.6 Histology of growth plate

Immunofluorescent staining was performed to investigate the distribution of Cathepsin K (CTSK, biomarker for actively resorbing osteoclasts) in the area around the femoral growth plate. Decalcified sections were treated with antigen retrieval solution, followed by a blocking step according to the manufacturer's instruction (Vector Elite ABC Kit, Rabbit PK-6101). Rabbit monoclonal anti-Cathepsin K antibody (1:100 dilution, ab314644; Abcam) was selected to indicate CTSK. Sections were incubated overnight at 4°C. Goat anti-Rabbit IgG (H + L) Cross-Adsorbed Secondary Antibody, Alexa Fluor™ 488 (1:200 dilution, A-11008; Invitrogen, Carlsbad, CA, USA) was used as the secondary antibody. VECTASHIELD Vibrance Antifade Mounting Medium with DAPI (H-1800; Vector Laboratories, Burlingame, CA, USA) was used as a counter stain and to mount the sections. The corresponding microscopic filter was used to visualize the stain.

For chondrocyte staining, freshly harvested femurs were dissected and fixed in 10% neutral buffered formalin at 4°C for 24 h. They were subsequently demineralized in ethylenediamine-tetraacetic acid (pH 7.3, Gojira Fine Chemicals, LLC) at 4°C for 28 days (n = 6 per group). The demineralized femora were then embedded in paraffin and sectioned into 5-μm-thick slices using a microtome (Epredia™ HM 355S Automatic Microtome). The sections were stained with Alcian blue and Safranin O (Electron Microscopy Sciences Co.) following the manufacturer's protocols to visualize the growth plate. Alcian blue staining was used to identify chondrocytes by binding to the acidic polysaccharides in cartilage (Goldstein and Horobin, 1974). In contrast, Safranin O staining, which binds to proteoglycan molecules, indicated the structural integrity and cellularity of the cartilage (Musumeci et al., 2014). The stained sections were examined and photographed under a light microscope (Imager.Z2; Zeiss).

Confocal microscopy was used to visualize growth plate chondrocytes as previously reported (Fernández-Iglesias et al., 2020). After harvesting, femurs were fixed with 2.5% glutaraldehyde for 24 h at 4°C. The undecalcified samples were then trimmed, stained with eosin Y (Sigma-Aldrich, 318906), embedded with epoxy resin under osmotically controlled conditions, and ground to 100 μm sections that were imaged with Nikon A1R confocal microscopy (Nikon Corporation, Japan).

### 2.7 Dynamic mechanical analysis (DMA) and static fracture testing

DMA and static fracture testing were conducted following previously established protocols (Kim et al., 2018; Liu et al., 2021). After non-destructive micro-CT scanning, the femurs were compressed at 55% of their length from the femoral head in the anterior-posterior direction using a 3-point bending jig with a 5 mm span length. An electromagnetic loading machine (Bose Corporation, Framingham, MA, USA) equipped with a displacement transducer (15 nm resolution) was utilized. Phosphate-buffered saline (PBS)-soaked paper towels were applied to keep the specimens moist throughout the experiment. Non-destructive bending oscillatory displacements of 0.01±0.005 mm were applied at frequencies of 0.5, 1, 2, and 3 Hz. Dynamic complex stiffness (K∗), elastic (storage) stiffness (K′), and viscous (loss) stiffness (K″), as well as tangent delta (tan δ) (K′′/K′), were measured. The tan δ accounts for energy dissipation capacity.

Following DMA testing, the same femur was subjected to compressive bending fracture at a displacement rate of 0.5 mm/s. The maximum force (F_max_), displacement at fracture (d_max_), and toughness (U) were determined from the load-displacement curve.

### 2.8 Statistical analysis

The Kolmogorov-Smirnov test was performed for all parameters in Table 1. Most parameters were not significant (p > 0.08), except Tt.Ar, I_min_, and GP Th.max (p < 0.42). These parameters were subsequently analyzed using the Mann-Whitney *U* test to compare the control (CON) and stress (RSD) groups. For the remaining parameters, a Student's t-test was used. Significance was set at p < 0.05.

**Table 1:** Comparison of measured parameters between the control (CON) and stress (RSD) groups (mean ± standard deviation) for the whole bone (WB), trabecular bone (TB), cortical bone (CB), and growth plate (GP) of femur. Significant (p < 0.05) differences are highlighted in **bold**.

Parameters		CON (n = 9)	RSD (n = 12)	p value
Physiology	**Spleen Weight (mg)**	**64.12±12.78**	**101.82±28.53**	**0.001**
	**Wound score**	**0**	**3.83±0.82**	**0.001**
	**IL-6 (pg/ml)**	**0.003±0.01**	**0.026±0.015**	**0.001**
	**TRAP (U/L)**	**6.10±0.72**	**7.26±0.65**	**0.03**
	**CTX-I (ng/ml)**	**12.82±1.22**	**18.15±2.65**	**0.01**
Volumetric	**TV (mm^3^)**	**37.25±1.57**	**35.31±1.85**	**0.01**
	**BV_WB_ (mm^3^)**	**19.01±1.39**	**17.74±1.17**	**0.04**
	**BV_CB_ (mm^3^)**	**14.51±9.24**	**13.56±9.92**	**0.03**
	BV_TB_ (mm^3^)	4.5±0.65	4.17±0.47	0.23
Mineral Density	BMD (mgHA/cm3)	529.38±30.02	506.69±33.18	0.11
	**TMC_WB_ (mgHA)**	**19.74±1.72**	**17.91±1.71**	**0.02**
	Mean_WB_ (mgHA/cm^3^)	1037.63±24.75	1008.31±47.40	0.08
	SD_WB_ (mgHA/cm^3^)	206.23±8.94	211.31±6.82	0.17
	**Low_5WB_ (mgHA/cm^3^)**	**685.01±27.86**	**637.33±42.4**	**0.005**
	High_5WB_ (mgHA/cm^3^)	1300.82±43.13	1288.24±68.87	0.61
	Mean_CB_ (mgHA/cm^3^)	1084.67±21.76	1058.34±43.87	0.08
	SD_CB_ (mgHA/cm^3^)	182.44±7.71	186.44±7.52	0.25
	**Low_5CB_ (mgHA/cm^3^)**	**767.69±33.5**	**716.47±38.34**	**0.004**
	High_5CB_ (mgHA/cm^3^)	1316.71±41.38	1303.93±69.16	0.60
	Mean_TB_ (mgHA/cm3)	881.49±40.06	846.88±58.39	0.12
	SD_TB_ (mgHA/cm3)	203.51±9.2	204.75±8.29	0.75
	**Low_5TB_ (mgHA/cm3)**	**581.4±34.11**	**529.82±56.77**	**0.01**
	High_5TB_ (mgHA/cm3)	1178.4±57.22	1155.01±67.61	0.40
Morphology	**Tt.Ar (mm^2^)**	**1.88±0.1**	**1.76±0.11**	**0.009**
	**Ct.Ar (mm^2^)**	**0.83±0.05**	**0.78±0.05**	**0.03**
	Ct.Ar/Tt.Ar	0.44±0.02	0.44±0.02	0.91
	Ct.Th (mm)	0.18±0.01	0.17±0.01	0.11
	**Perimeter (mm)**	**5.39±0.16**	**5.21±0.14**	**0.01**
	D_AP_outer_ (mm)	1.27±0.05	1.24±0.05	0.14
	D_ML_outer_ (mm)	1.90±0.07	1.75±0.27	0.09
	D_AP_inner_ (mm)	0.92±0.05	0.99±0.27	0.41
	D_ML_inner_ (mm)	1.44±0.08	1.14±0.06	0.31
	AP/ML	0.66±0.02	0.73±0.2	0.28
	**I_min_ (mm^4^)**	**0.12±0.01**	**0.1±0.01**	**0.03**
	**BV/TV_TB_**	**0.37±0.09**	**0.27±0.04**	**0.01**
	BS/BV (mm^−1^)	53.71±9.32	58.1±5.83	0.23
	**Tb.N (mm^−1^)**	**5.46±0.55**	**4.23±0.52**	**<0.001**
	Tb.Th (mm)	0.06±0.01	0.06±0.00	0.61
	**Tb.Sp (mm)**	**0.12±0.01**	**0.15±0.01**	**<0.001**
Growth Plate (GP)	**TV_GP_ (mm^3^)**	**1.08±0.07**	**0.98±0.07**	**0.01**
	BV_GP_ (mm^3^)	0.79±0.05	0.74±0.05	0.09
	BV/TV_GP_	0.73±0.05	0.76±0.03	0.19
	GP Surface area (mm^2^)	0.2±0.05	0.2±0.05	0.81
	**GP Th.mean (mm)**	**0.07±0.00**	**0.06±0.01**	**0.01**
	GP Th.max (mm)	0.12±0.01	0.11±0.02	0.24
	Mean_GP_ (mgHA/cm^3^)	508.09±53.43	504.54±36.01	0.86
	**SD_GP_ (mgHA/cm^3^)**	**207.40±4.17**	**212.13±5.92**	**0.04**
	**Low_5GP_ (mgHA/cm^3^)**	**258.01±42.184**	**215.68±52.3**	**0.05**
	High_5GP_ (mgHA/cm^3^)	933.36±43.76	916.99±36.01	0.37
Dynamic mechanical analysis (DMA)	K∗ (N/mm)	153.15±36.93	133.35±19.8	0.17
	K′ (N/mm)	152.93±36.86	133.16±19.8	0.17
	K′′ (N/mm)	7.92±2.59	6.85±1.11	0.27
	tan δ	0.05±0.009	0.05±0.009	0.86
Fracture	**F_max_ (N)**	**18.21±3.43**	**12.26±2.37**	**0.01**
	d_max_ (mm)	0.29±0.09	0.26±0.09	0.93
	U (Nmm)	3.52±1.72	2.16±1.09	0.37

## 3 Results

To determine how RSD might affect bone quality, 3D micro-CT scanning was performed on femurs, and analysis was performed on whole bone (WB), the dense cortical bone (CB) composing the shaft of the femurs, and the internal trabecular bone (TB). Fig. 1a demonstrates how these compartments were digitally isolated from images of whole bone. Frequency plots of tissue mineral density (TMD) were then generated to determine how stress might affect bone quantity. Fig. 1b–d showed that the Low_5_ values of each femoral region (Low_5WB_, Low_5CB,_ Low_5TB_) in the stress (RSD) group were significantly lower than those in the control (CON) group (p < 0.01), resulting in a significant reduction in the total mineral content (TMC) of the WB in the stress group (Table 1 and Fig. 1e–h). These findings represent a diminished bone content in femurs of RSD mice relative to non-RSD controls.

**Fig. 2:**
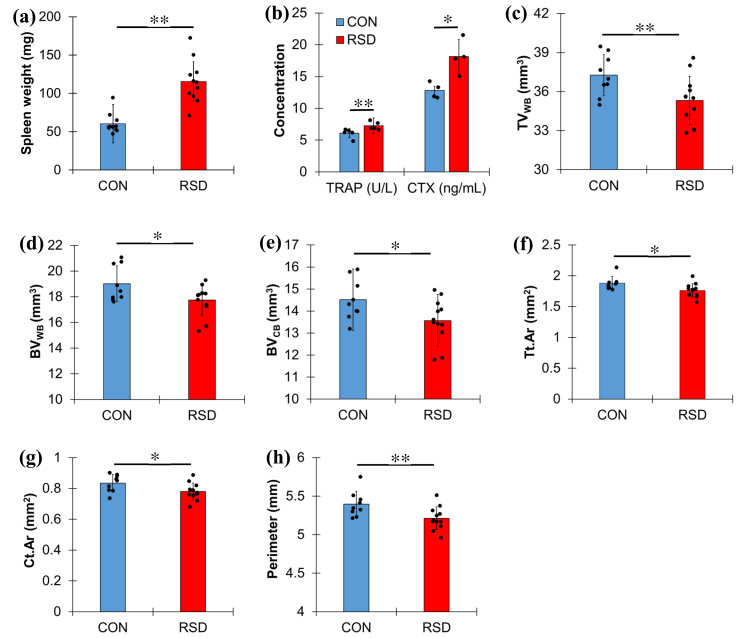
Significant differences in parameters between control (CON) and stress (RSD) groups for (a) spleen weight, (b) TRAP (tartrate-resistant acid phosphatase) and CTX-I (carboxyterminal cross-linking telopeptide of bone collagen) concentrations in serum, and (c) total volume (TV_WB_) and (d) bone volume (BV_WB_) of the WB, as well as (e) bone volume (BV_CB_), (f) total area (Tt.Ar), (g) cortical area (Ct.Ar), and (h) perimeter of the CB. ∗; p < 0.05, ∗∗; p < 0.01.

The wound score was significantly higher in RSD mice than in control mice (p < 0.001). Spleen weight and IL-6, which are established biomarker of the stress response, were significantly greater in the RSD group compared to the control group (p < 0.001), demonstrating the success of the stress treatments (Table 1 and Fig. 2a). This increased weight is indicative of heightened myelopoiesis in the bone marrow and mobilization of these cells to the spleen (McKim et al., 2018b). Osteoclasts, derived from myeloid precursors, were also present and actively resorbing bone at higher numbers in the RSD groups than controls, as indicated by serum measurements of TRAP and CTX-I (p < 0.03) (Fig. 2b). As a result, femurs from the RSD group had significantly smaller volumes (TV_WB_, BV_WB_, BV_CB_) than the control group (p < 0.04) (Fig. 2c–e), due to a significant decrease in CB morphology (Tt.Ar, Ct.Ar, and perimeter) in the stress group (p < 0.03) (Fig. 2f–h).

**Fig. 3:**
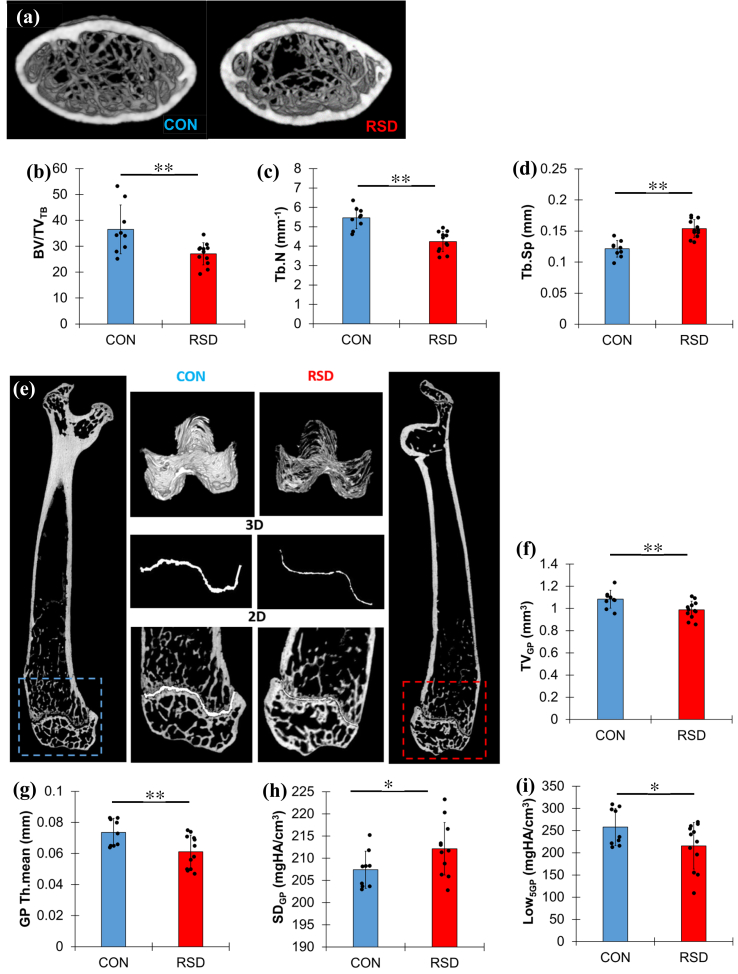
Micro-CT based comparisons of (a) trabecular bone (TB) architecture between control (CON) and stress (RSD) groups. Significant differences in the TB and GP parameters between control (CON) and stress (RSD) groups for the TB (b) bone volume fraction (BV/TV_TB_), (c) trabecular number (Tb.N), and (d) trabecular separation (Tb.Sp). (e) Growth plate (GP) locations, volume, and thickness were identified. Significant differences of GP (f) total volume (TV_GP_), (g) mean thickness (GP Th.mean), (h) tissue mineral density (TMD) standard deviation (SD_GP_), and (i) 5th percentile low (Low_5GP_). ∗; p < 0.05, ∗∗; p < 0.01.

**Fig. 4:**
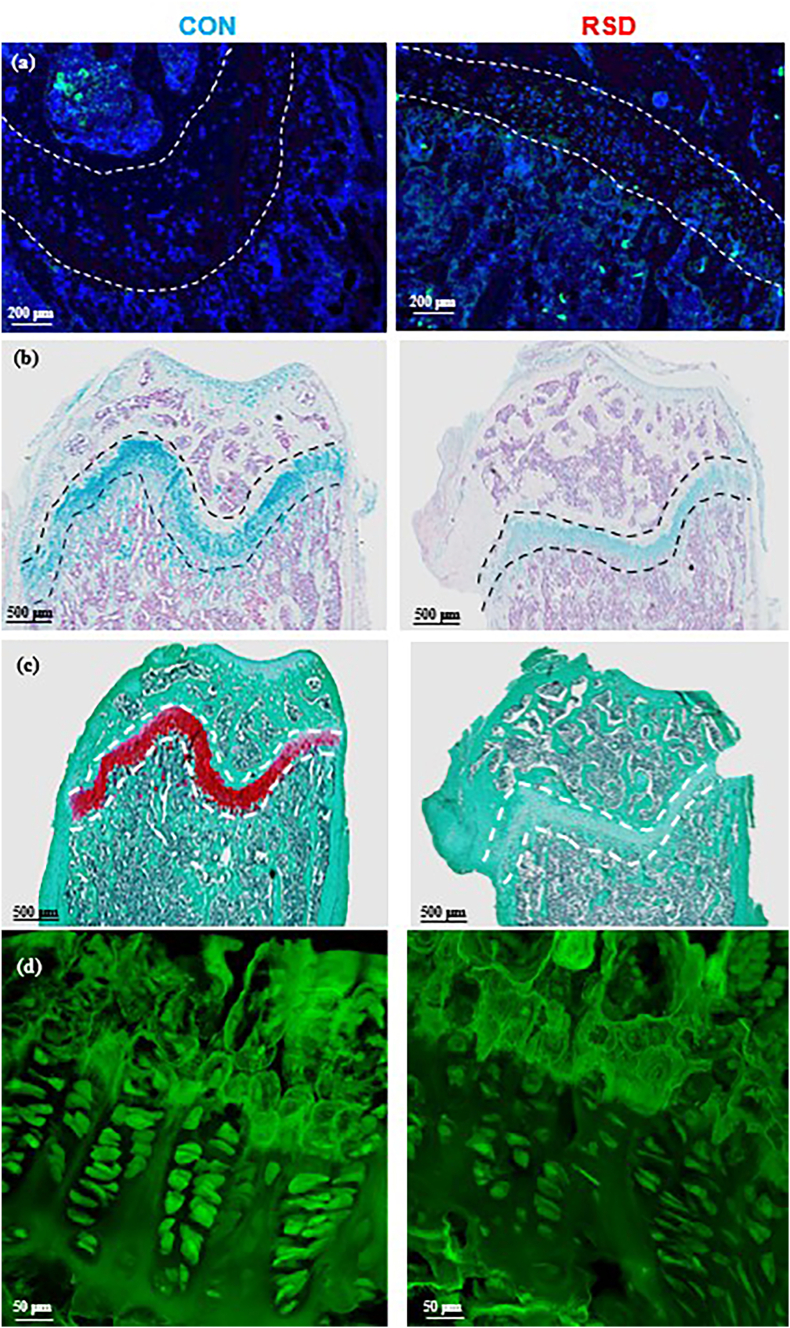
(a) Immunohistochemistry revealed CTSK (green) in the stress group (RSD) but not in the control group (CON). (b) Alcian blue and (c) Safranin O staining of the cartilage matrix in distal growth plate showed significantly lighter or no staining in RSD. (d) Confocal microscopy showed 3D visualization for less organized stack of chondrocytes in RSD than CON growth plate. (For interpretation of the references to colour in this figure legend, the reader is referred to the Web version of this article.)

The TB architecture (BV/TV_TB_, Tb.N, and Tb.Sp) was also significantly decreased in the RSD group compared to the control group (p < 0.01) (Fig. 3a–d). Further, because severe psychological stress was previously linked to short stature in human adolescents (Mousikou et al., 2023), we examined the structure of the femoral distal growth plate (GP) (Fig. 3e). The total volume (TV_GP_) and mean thickness (GP Th.mean) significantly decreased in the stress (RSD) group relative to the control group (p < 0.01) (Fig. 3f and g), indicating potential growth plate dysfunction. The TMD Low_5GP_ was also reduced, leading to an increase in heterogeneity (SD_GP_) (Fig. 3h and i). Immunohistochemistry revealed that the osteoclast biomarker (CTSK) increased in the GP of the RSD group (Fig. 4a), whereas multiple stains for chondrocyte activity indicated less activity in the GP of the RSD group compared to the control group (Fig. 4b, c, and d).

**Fig. 5:**
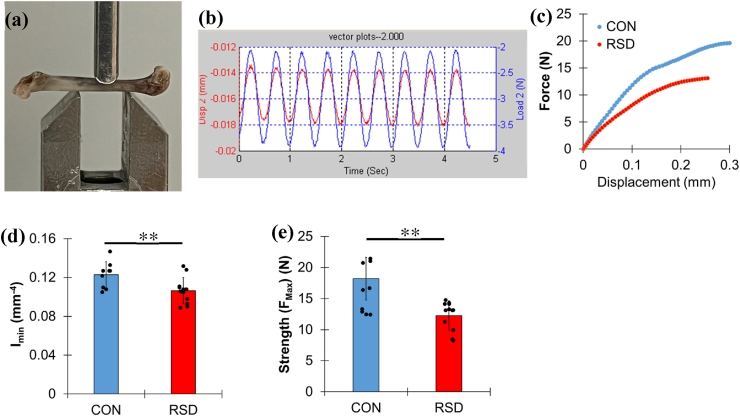
(a) 3-point bending test at 55% of the femoral length from the head, (b) dynamic mechanical analysis (DMA) signal under non-destructive cyclic bending displacement (−0.01±0.005 mm), (c) force-displacement curve from static fracture testing, Significant differences in the cortical bone (CB) parameters between control (CON) and stress (RSD) groups for (d) minimum inertia (I_min_) and (e) femoral bending strength, measured as the maximum force (F_Max_) from the fracture curve. ∗∗; p < 0.01.

Non-destructive bending oscillatory displacements were applied at 55% of the femoral length from the head (Fig. 5a and b) and fracture testing was followed (Fig. 5c). The decreased CB morphological changes resulted in smaller inertia (I_min_) in the stress group, leading to weaker femur strength compared to the control group (p < 0.01) (Fig. 5d and e).

All other measured parameters showed no significant differences between the stress and control groups (p > 0.08) (Table I).

## 4 Discussion

The repeated social defeat (RSD) mouse model in adolescent males showed significantly increased spleen weights and stimulated osteoclastic activity, as indicated by significantly elevated biomarkers of bone resorption (TRAP and CTX-I) in the stress group. The increased bone resorption significantly reduced tissue mineral, volume, and morphology of femur, resulting in diminished bone strength and resistance to fracture. In addition, the distal femoral growth plate was significantly thinner in the RSD group than the control group and showed higher osteoclast but less chondrocyte activities. These changes in bone quantity and quality illustrate how severe repeated psychosocial stress in young mice, even over a short time frame (6 days), can be a powerful mediator of bone health.

Spleen weight, an established biomarker of successful RSD and the resulting enhanced monocytopoiesis, was significantly higher in the stress group compared to the control group. IL-6, a pro-inflammatory cytokine that promotes the proliferation of myeloid lineage cells including monocytes, was also increased in the RSD group. These results are consistent with findings from previous RSD mice studies (Niraula et al., 2018; Weber et al., 2017; Yin et al., 2019; Sawicki et al., 2019; Kinsey et al., 2008) validating the current RSD model. The increased spleen weights of the RSD mice demonstrate increased monocyte mobilization and an ongoing stress response, as shown in previous studies (Niraula et al., 2018; Weber et al., 2017; Kinsey et al., 2008). Briefly, RSD activates the sympathetic nervous system and the hypothalamic-pituitary-adrenal axis to mobilize Ly6C^hi^ monocytes from the bone marrow to the spleen, resulting in increased splenic mass. The spleen then acts as a reservoir to allow distribution of primed and glucocorticoid-insensitive monocytes throughout the body, including the central nervous system, where accumulation of monocytes results in increased anxiety- and depressive-like behaviors.

Additionally, stressed mice had significantly elevated levels of serum TRAP (p = 0.03) as well as markedly higher levels of CTX-I (p = 0.01). Serum TRAP, secreted by osteoclasts, reflects osteoclast activity, while CTX-I, a degradation product of type I collagen, results from osteoclastic bone resorption. As such, these findings directly demonstrate the enhancing effects of RSD on osteoclast number and activity, leading to substantial bone resorption.

Osteoclasts are activated to initiate bone resorption, followed by osteoblast-mediated bone formation, which together drive the process of bone remodeling. When these coupled activities of bone cells are balanced, the quantity of bone is maintained, while the composition (quality) of bone tissue is changed as pre-existing mineralized bone tissues are removed and the newly formed, less mineralized bone tissues are added. Following the primary mineralization, a long-term secondary mineralization gradually progresses (Ruffoni et al., 2007; Roschger et al., 2008). This bone turnover rate is estimated to be approximately 4.25 months for humans, including 30 days of bone resorption and 80 days of bone formation and mineralization. In comparison, this rate is about 3 months for dog, 1.5 months for rabbit, and 0.5 months (2 weeks) for mouse (Martin et al., 1998; Brunski, 1999; Roberts et al., 1987; Jilka, 2013). As the mouse femurs used in the current study were dissected within 0.5 days after 6 days of RSD, these are estimated at the primary mineralization stage of newly formed bone following bone resorption. The current findings support this, as the tissue mineral contents and Low_5_ values of each bone regions were significantly lower in the stress group than in the control group.

Bone loss occurs only if bone resorption is not coupled with bone formation during bone modeling, or if the resorbed bone is not completely filled with new bone during remodeling. This imbalance in bone modeling and remodeling is a key factor in the development of osteoporosis (Seeman, 2003; Eastell et al., 2016). The current study observed significant bone loss in the TB of the stress group, showing lower trabecular number and bone fraction but more surface area than that of the control group. The porous trabecular network provides larger surfaces of bone providing a higher chance to recruit more bone cells. As such, the removal of tissue at the TB surfaces and the eventual disconnection of the TB network have been accepted as the first indication of events that could occur in early osteoporosis (Osterhoff et al., 2016).

Maturation of the growth plate involves the replacement of cartilage with new trabecular bone formation, resulting in narrow cartilaginous zones (Yakar et al., 2002; Romeo et al., 2019; Staines et al., 2018). In the current study, RSD-induced alterations to the growth plate were visualized and quantified in adolescent male mice. The micro-CT-based 3D image analysis revealed that the growth plates in the RSD group had significantly smaller thickness and volume. This result is likely due to the addition of newly formed, less mineralized bone tissue to the growth plate, leading to reduced TMD Low_5GP_ and increase in heterogeneity (SD_GP_). Furthermore, immunohistochemistry revealed the biomarker for osteoclasts in the RSD growth plate (Fig. 4a), and cationic dyes that stain the extracellular matrix produced by chondrocytes showed much lower staining in the RSD samples compared to the control group, confirming alterations in chondrocyte function (Fig. 4b and c). Finally, confocal microscopy showed that stacks of chondrocytes were less organized in RSD than control growth plate (Fig. 4d). These findings are consistent with well-established research in human children and adolescents, where abusive environments and emotional deprivation are linked to short stature (Mousikou et al., 2023).

Bone apposition occurs on the periosteum, while both bone resorption and formation are observed on the endosteum of the mouse femur (Scheuren et al., 2017). The stressed group showed significantly smaller bone area and perimeters of the CB than the control group. However, there was no significant difference in CB thickness between the groups. This finding suggests that RSD inhibits periosteal bone apposition, while endosteal bone formation continues. These morphological changes decreased the ability of femoral cross-sections to resist bending (minimum inertia (I_min_)), leading to significant reduction of its strength. Although dynamic mechanical properties were also decreased, the changes were not statistically significant. These results indicate that RSD could increase the fracture risk of bone.

RSD is one of a variety of psychological stress models that have been developed and characterized in recent years. Current murine models include acute stressors such as single episode restraint or underwater trauma, as well as chronic stressors such as social defeat or chronic unpredictable mild stress, also known as chronic variable stress (Gyles et al., 2024). These models result in changes to the hypothalamic-pituitary-adrenal (HPA) axis and the sympathetic nervous system (SNS), though the precise outcomes appear to vary with the type of psychological stress administered. RSD activates both the HPA and SNS systems, leading to increased levels of corticosteroids and catecholamines (Weber et al., 2017). Both systems have the potential to influence bone cell activity, though the SNS is best established to play a role in bone homeostasis (Shi and Chen, 2024). Both osteoblasts and osteoclasts bear beta-adrenergic receptors, and signaling through these receptors inhibits osteoblastic bone formation while enhancing osteoclastic bone resorption. In addition, pro-inflammatory mediators are potent activators of osteoclast formation and activity (Terkawi et al., 2022). The relative contributions of inflammation and SNS activation to enhanced osteoclast activity in RSD are as yet unknown and will require further examination.

Previous studies of bone homeostasis in models of psychological stress appear to indicate that the duration and frequency of stress play key roles in which physiological systems are activated. For example, the chronic subordinate colony housing paradigm (CSC), in which young mice are continually housed with a dominant mouse for 19 days, results in activation of the SNS but not the HPA axis (Reber et al., 2007). The long bones of these mice were found to be shortened, but unlike the RSD model, CSC resulted in increased growth plate thickness and increased trabecular bone mineral density without changes in osteoblast and osteoclast numbers, potentially indicating chondrocyte dysfunction (Foertsch et al., 2017). In contrast, a recent study in which adolescent mice were subjected to intermittent variable stressors, the chronic unpredictable mild stress (CUMS) model (Sharma et al., 2024), revealed loss of trabecular and cortical bone, a slight increase in bone formation, but a significantly enhanced increase in osteoclast activity (Zhang et al., 2025). Although the stressors in the CUMS paradigm are broad in nature (e.g. restraint, water immersion, social stress, circadian rhythm disruption, among others), this model has similarities to RSD in the application of intermittent stresses, resulting in HPA and SNS dysfunction, neuroinflammation, bone loss, and osteoclast activation. Interesting recent developments have linked psychological stress to miRNAs that produce bone loss via activation of osteoclasts. In the aforementioned CUMS study, miR-335-3p was shown to be downregulated, leading to increased osteoclast differentiation and activity (Zhang et al., 2025), while other studies revealed that sympathetic stimulation in mice triggered synthesis of miR-21 in osteoblasts, which is subsequently transferred to osteoclast progenitors via exosomes to increase osteoclastogenesis (Hu et al., 2022). Future examination of how gene and miRNA expression may be altered in RSD mice could reveal further similarities and differences with other social stress models.

A limitation of this study is the short period of experiments to collect bone right after 6 days of RSD. Given that the typical lifespan of an osteoclast is approximately 2 weeks (Jilka, 2013), it is likely that bone loss resulting from increased osteoclastic activity induced by RSD would be maintained or even exacerbated over a longer period following the stress. In addition, our previous studies showed that some elements of RSD-induced monocyte trafficking remain elevated 8 days after RSD, though these disappeared by 24 days post-RSD (Wohleb et al., 2014). Accordingly, we expect osteoclast activity to be maintained for a limited period of up to 8 days post-RSD, leading to increased bone loss and growth plate reduction. Further studies are needed to assess bone quantity and quality at extended time periods, such as 8- and 24-days post-RSD.

Another limitation of this study is that the current experiments were performed only on male mice. It is well-established that sex differences in neuro-immune responses and behavior exist as a consequence of social stress; however, male mice do not naturally demonstrate aggressive behavior toward females of reproductive age, preventing our ability to use the current protocols to test responses in females. Nonetheless, we previously demonstrated that genetically modified DREADD male mice can be induced to elicit male aggression toward females (Yin et al., 2019). In these studies, stressed females demonstrated anxiety-like behavior and inflammatory responses similar to those of males, including increased spleen weight due to monocyte trafficking, elevated IL-6 levels, and monocyte accumulation in the brain. Thus, we expect that bone loss due to increased inflammation and osteoclastogenesis is a likely outcome in females, though further experimentation will be required to confirm this expectation.

In conclusion, we found that 6 days of RSD were sufficient to produce significant and immediate bone loss in the femur of adolescent male mice. RSD enhanced osteoclast activity, leading to the removal of bone tissue from the bone surface, resulting in bone loss. As new bone formation is coupled with bone resorption, less mineralized new bone tissues are added to the resorbed sites. The TB of RSD group is removed and disconnected because the larger surface area of TB recruits more bone cells for active bone resorption. Additionally, RSD likely inhibited periosteal bone formation in the femur of adolescent mice, as evidenced by smaller CB perimeters, which contributed to a substantial decrease in femoral fracture strength. The growth plate was also thinner and more disorganized in the stress group. These results provide insight into the central role of osteoclasts in the kinetics of RSD-induced alterations of bone and provide a paradigm for further examination of how this type of stress may affect skeletal health for animals of different ages and sex.

## Declaration of generative AI and AI-assisted technologies in the writing process

During the preparation of this work the author(s) used ChatGPT-40 mini in order to check the grammar and edit text for clarity during the manuscript's preparation. After using this tool/service, the author(s) reviewed and edited the content as needed and take(s) full responsibility for the content of the publication.

## CRediT authorship contribution statement

**Do-Gyoon Kim:** Conceptualization, Data curation, Formal analysis, Funding acquisition, Investigation, Methodology, Project administration, Resources, Software, Supervision, Validation, Visualization, Writing – original draft, Writing – review & editing. **Farah A. Al-Omari:** Data curation, Formal analysis, Investigation, Methodology, Validation, Visualization, Writing – original draft, Writing – review & editing. **Natalie R. Gallagher:** Data curation, Formal analysis, Investigation, Methodology, Project administration, Resources, Validation, Visualization, Writing – original draft, Writing – review & editing. **Jiyeon Kim:** Data curation, Investigation, Methodology, Validation, Visualization, Writing – original draft, Writing – review & editing. **Jie Liu:** Data curation, Investigation, Methodology, Validation, Visualization, Writing – original draft, Writing – review & editing. **Minji Kim:** Data curation, Investigation, Methodology, Validation, Visualization, Writing – original draft, Writing – review & editing. **Olivia Jackson:** Data curation, Investigation, Methodology, Validation, Visualization, Writing – original draft, Writing – review & editing. **John F. Sheridan:** Conceptualization, Formal analysis, Funding acquisition, Investigation, Project administration, Resources, Supervision, Validation, Visualization, Writing – original draft, Writing – review & editing. **Beth S. Lee:** Conceptualization, Data curation, Formal analysis, Funding acquisition, Investigation, Methodology, Project administration, Resources, Software, Supervision, Validation, Visualization, Writing – original draft, Writing – review & editing.

## Declaration of competing interest

There are no conflicts of interest for any author.

## Data Availability

Data will be made available on request.
